# Task-Specific Effects of mGlu2/3 Receptor Agonist LY379268 on MK-801-Induced Behavioral and Neural Dysfunctions in Rats

**DOI:** 10.33549/physiolres.935715

**Published:** 2026-02-01

**Authors:** Karolina HRUZA, Daniela CERNOTOVA, Kristyna MALENINSKA, Jan SVOBODA, David LEVCIK, Ales STUCHLIK

**Affiliations:** 1 Laboratory of the Neurophysiology of Memory, Institute of Physiology of the Czech Academy of Sciences, Prague, Czech Republic; 2 Third Faculty of Medicine, Charles University, Prague, Czech Republic; 3 National Institute of Mental Health, Klecany, Czech Republic

**Keywords:** MK-801, LY379268, Electrophysiology, Medial prefrontal cortex, Hyperlocomotion

## Abstract

NMDA receptor hypofunction can lead to behavioral and cognitive disturbances, including hyperlocomotion, and is considered a core pathophysiological mechanism underlying cognitive and negative symptoms in schizophrenia. This study examined whether treatment with the mGlu2/3 receptor agonist LY379268 (1 and 2 mg/kg) could counteract such disruptions induced by the NMDA antagonist MK-801 (0.1 mg/kg). Rats were tested under two conditions: an aversive learning task (active place avoidance on a rotating arena) and a non-aversive open field test. Additionally, local field potentials were recorded from the medial prefrontal cortex during the open field test and later under urethane anesthesia. Contrary to expectations, LY379268 did not consistently alleviate MK-801-induced impairments. In the aversive learning context, the combination of MK-801 with LY379268 (2 mg/kg) paradoxically led to exacerbated hyperlocomotion and impaired navigational performance. In contrast, the 1 mg/kg dose of LY379268 had a modest beneficial effect in the non-aversive setting, slightly reducing MK-801-induced hyperactivity. Electrophysiological recordings revealed that MK-801, alone or in combination with LY379268 (1 mg/kg), disrupted theta-high gamma phase-amplitude coupling in the open field test, indicating impaired neural processing. Under anesthesia, MK-801 increased low gamma power. LY379268 did not reverse this alteration. These findings highlight the task- and dose-dependent nature of LY379268’s effects. While it offered limited improvement in a non-aversive environment, it failed to mitigate and sometimes exacerbated deficits in more challenging, aversive tasks. This complexity underscores the need for further research to refine the therapeutic potential of mGlu2/3 modulation in conditions associated with glutamatergic dysfunction.

## Introduction

Cognitive deficits encompass a range of complex symptoms arising from impairments in mental processes essential for proper daily functioning. These impairments can manifest as deficits in memory, attention, decision-making, spatial navigation, and various other cognitive processes. Such deficits are prevalent across numerous psychiatric conditions, including schizophrenia, bipolar disorder, attention-deficit/hyperactivity disorder (ADHD), obsessive–compulsive disorder (OCD), depression, and anxiety-related disorders [1]. Among these conditions, schizophrenia stands out as a particularly severe mental disorder, affecting approximately one percent of the general population with an increasing incidence [2,3]. A leading hypothesis for the etiology of schizophrenia centers on the N-methyl-D-aspartate (NMDA) receptor hypofunction [4]. Non-competitive antagonists of NMDA receptors, such as ketamine, phencyclidine (PCP), and particularly dizocilpine (also known as MK-801), are commonly employed in primary research to induce cognitive deficits that result in psychosis-like behavior. This approach is frequently used to create animal models of schizophrenia, mainly to mimic both positive and negative symptoms [5].

MK-801 primarily affects the firing rate of γ-aminobutyric acid (GABA) interneurons [6]. As a highly potent NMDA receptor antagonist, it binds more persistently than ketamine or PCP, providing more substantial channel-blocking effects [7]. Most cells affected by MK-801 are parvalbumin-positive (PV+) interneurons, which can be distinguished by their unique electrophysiological properties [8]. The reduced activity of PV+ interneurons decreases signal-to-noise ratio and disrupts the excitation/inhibition (E/I) balance by elevating the firing rate of pyramidal cells and increasing glutamate release due to reduced inhibition from PV+ interneurons [9]. Consequently, this alteration impacts high-frequency oscillations [10] and mainly disrupts theta-gamma coupling [11] a phenomenon also observed in patients with schizophrenia [12].

In our previous research, we showed that optogenetic stimulation of PV+ interneurons in the medial prefrontal cortex (mPFC) mitigated the negative impact of MK-801 on cognitive flexibility [13]. The prefrontal cortex (PFC) is a key region implicated in the pathophysiology of schizophrenia, particularly in relation to cognitive dysfunction [14]. Evidence for this connection comes from multiple sources: firstly, non-schizophrenic patients with prefrontal lesions exhibit schizophrenia-like symptoms, particularly from the negative and cognitive spectrum [15]. Secondly, patients with schizophrenia have decreased metabolism, decreased blood flow, and impaired function of the PFC [16]. An imbalance in the PFC signaling induces hyperlocomotion and may contribute to the positive symptoms of schizophrenia [17]. Another symptom of schizophrenia includes psychomotor agitation, character-rized by increased psychomotor activity and motor restlessness [18]. Hyperlocomotion can be modeled in animals using NMDA receptor antagonists like MK-801, which primarily induces hyperactivity at low doses (~0.1 mg/kg), resembling psychosis-like behavior [19].

A promising approach for alleviating symptoms associated with increased glutamatergic release in psychosis-like behaviors is targeting mGlu2/3 receptors. These presynaptic inhibitory autoreceptors reduce excitatory glutamate transmission by decreasing glutamate release, thereby preventing glutamate-induced hyperexcitability and maintaining physiological glutamate levels through a negative feedback mechanism [20]. Group II metabotropic glutamate receptor (mGluR) agonists have emerged as protective agents against hyperexcitability, helping to sustain E/I balance and suggesting potential antipsychotic effects by countering excessive excitation. Among these, LY379268 has shown promise as a therapeutic candidate for treating psychosis [21]. Systemic administration of LY379268, rather than a localized injection, effectively reduces glutamatergic overactivation, indicating that mGluRs contribute to this effect in brain regions projecting to the mPFC [22]. Additionally, LY379268 has demonstrated efficacy in reducing hyperlocomotion, further supporting its potential in modulating psychosis-like behaviors [23]. However, although mGlu2/3R agonists (for example, pomaglumetad methionil) showed promising preclinical results, clinical trials in schizophrenia yielded mixed outcomes, possibly due to dosing or mechanistic uncertainties [24].

In this study, we explore the potential therapeutic-like effects of systemic administration of LY379268 on MK-801-induced psychosis-like behaviors, focusing on spatial navigation in an aversive rotating arena task and spontaneous behaviors in non-aversive open field test. We hypothesize that MK-801 administration will increase locomotion, which will be mitigated by pretreatment with LY379268. We also aim to determine whether the NMDA antagonist MK-801 leads to desynchronization in local field potentials (LFP) within the mPFC, a disruption associated with cognitive and behavioral deficits. Additionally, we investigate whether pretreatment with LY379268 can offer protective effects by attenuating LFP desynchronization, thereby helping to maintain neural synchrony in the mPFC. Together, these analyses will provide insight into the efficacy of LY379268 in counteracting the behavioral and neural consequences of NMDA receptor antagonism. We anticipate that MK-801 will induce increased gamma oscillations and reduce synchronization between theta and gamma oscillations. These effects are expected to be attenuated by pretreatment with LY379268.

## Materials and Methods

### Animals

We used 83 Long Evans male rats (breeding core obtained at Charles River, rats produced in a breeding colony of the Institute of Physiology of the Czech Academy of Sciences) aged 3–4 months and weighing 250–350 g. The rats were housed in pairs in transparent plastic cages in standard and controlled conditions (22 °C, 50–60 % humidity, 12-hour light/dark cycle). For the rotating arena test, 57 of these animals were used, divided into the following groups: saline controls (n=11), MK-801 (0.1 mg/kg) group (n=15), and 2 groups with combined LY379268 and MK-801 administration at two different doses (n=13 with LY379268 1 mg/kg dose, n=17 with LY379268 2 mg/kg dose). One animal from the MK-801 group was excluded due to hypersensitivity to MK-801. Animals were tested in four runs, with a group of 14 animals per run; each run included all four experimental groups.

A second cohort (n=26) was used for electrophysiological recordings. The rats were divided into the following groups: saline controls (n=7), MK-801 (n=7), LY379268 (n=7; 1 mg/kg dose), and a combined treatment of MK-801 and LY379268 (n=5). After exclusion of animals with incorrect electrode placement, the final sample sizes used in the electrophysiological analyses were: saline (n=7), MK-801 (n=5), and LY379268 + MK-801 (n=5); the LY379268-only group included three valid recordings and was therefore not included in the main electrophysiological comparisons. One week before the experiment, the animals were food-restricted, maintained at 85–90 % of their body weight, and they were handled for 5 min per day for 5 days. Mild food restriction was employed to minimize variability in locomotor and exploratory behavior, as this cohort of animals exhibited more rapid weight gain compared to the previous one, which could have influenced pharmacokinetics and potentially impacted the pharmacological responses.

All animal treatments were approved by the Local Animal Care Committee (51-2022-P) and complied with the Animal Protection Code of the Czech Republic and the European Community Council directive (2010/63/EC). Maximum efforts were made to minimize animal suffering, emphasizing the 3Rs principle.

### Stereotaxic surgeries

For the electrophysiological experiment, 24 rats were implanted with recording electrodes bilaterally into the medial prefrontal cortex (mPFC). The custom-made implant consisted of two silver wire electrodes (0.254 mm diameter; A-M Systems, USA) soldered to Mill-Max connector, with one ground/reference screw. The electrodes were used to record local field potentials (LFP) in the mPFC of both hemispheres. One week before surgery, rats were treated with antibiotics dissolved in their drinking water (enrofloxacin, 100 mg/ml) to prevent infections.

Anesthesia was induced with 5 % isoflurane and maintained at 1.2 % to 2.5 % during the surgeries, depending on the individual animal. The electrodes were inserted into the brain through small, drilled holes in the skull at coordinates corresponding to the mPFC region (AP=+3 mm, ML=±0.7 mm, DV=4 mm from bregma, according to the brain atlas; (31). One ground screw, which also served as a reference, was placed above the cerebellum, and two anchoring screws were placed posterior to the electrodes. The implant was then attached to the screws and skull with methacrylate resin. The incision was sutured, and the skin was glued with biocompatible glue (Surgibond) and locally injected with mesocaine (10 mg/ml). Shortly before awakening, rats were treated subcutaneously with 10 % carprofen (50 mg/kg).

Rats with electrophysiological implants were kept in pairs in larger plastic cages (55×35×27 cm) to minimize the risk of implant damage. Antibiotics (enrofloxacin, 100 mg/l) and analgesics (ibuprofen, 100 mg/l) were administered in the drinking water for five days after the surgery. Rats were monitored daily for their health status and allowed to recover completely for at least two weeks before the experiments began.

### Drugs and treatments

We followed the same administration scheme for the behavioral and electrophysiological experiments. All drugs were injected intraperitoneally. The control group received a saline injection (0.9 % NaCl, 1 ml/kg) 30 min before the experiment, identical to the MK-801-treated group (MK-801, Sigma-Aldrich, 0.1 mg/kg, Cat. No. M107, diluted in saline). LY379268 (Tocris, Cat. No. 2453) was administered 1 h before testing at either 1 mg/kg or 2 mg/kg, dissolved in saline. For the combination treatment, animals received LY379268 1 h and MK-801 30 min before the testing (LY379268 + MK-801 groups). For avoidance learning on the rotating arena, we used dosing of 1 mg/kg and 2 mg/kg of LY379268. For the electrophysiological recordings, a dose of 1 mg/kg LY379268 was used.

### Behavioral testing

#### Active place avoidance on the rotating arena

**Fig. 1:**
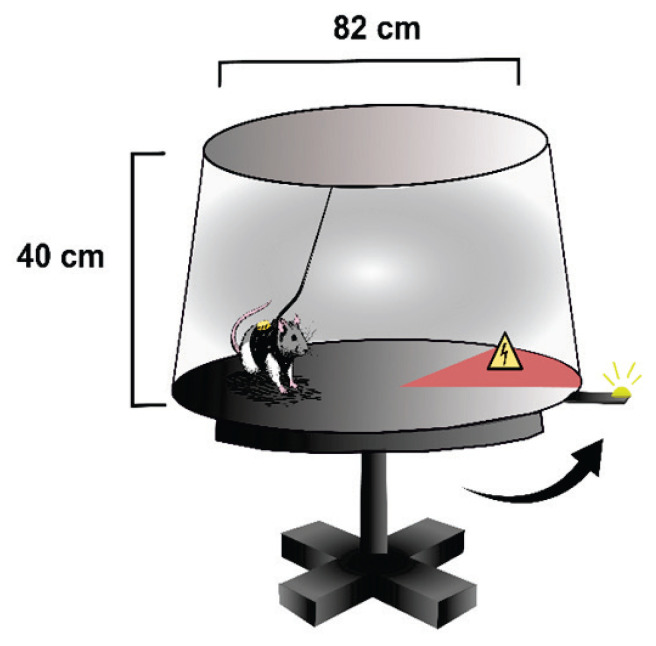
Schematic illustration of the rotating arena apparatus with the rat. The rats avoided an unmarked 60-degree sector, entering which was punished by a mild footshock.

The active place avoidance task is a spatial learning test conducted in a rotating metallic arena (82 cm in diameter), also known as the Carousel maze, which is enclosed by a 40-cm-high transparent Plexiglas wall and elevated 1 m above the floor; in this setup, animals must actively avoid a designated location by relying on external visual cues for orientation (Fig. 1). A day before the experiment, a needle was pierced through a skin fold between the shoulders to allow shock delivery throughout the sessions, with the tip bent to prevent slipping out. This procedure resembled the subcutaneous injections; thereby, it did not require anesthesia. The animals were habituated to wear a small harness with a tracking infrared LED and grounding attached to the needle for receiving mild electrical shocks (0.4 mA). On the first day of habituation, the animals wore the tracking vest and were placed in the stable arena without receiving shocks, allowing them to explore freely for 10 min. During the learning phase (days 2–6), the arena was rotating constantly at one revolution per minute. An unmarked 60-degree sector was designated as a to-be-avoided zone. The sector remained stable relative to the room, requiring the animal to actively avoid it by using room-stable spatial cues while disregarding the misleading arena-based cues. Each daily session lasted for 20 min. The animal received 0.4 mA footshocks with 900 ms intervals between shocks to motivate it to leave the to-be-avoided sector. Rat movement was monitored using Tracker, Biosignal Group, USA. Recordings were analyzed with an open-source Carousel Maze Manager 0.5.0 [25]. The apparatus is described in more detail elsewhere [26,27].

### Open field test

Open field test was used for the evaluation of exploratory behavior. We used a squared white open field arena enclosed with white walls (70 cm×70 cm×50 cm), illuminated to 36 lx in the center for the open field test. After drug administration, the animals were connected to a recording cable and placed in the center of the open field arena. Their behavior was recorded using an overhead camera, and the footage was later analyzed offline. The rats were allowed to explore freely for 15 min. Distance traveled and time spent in the center of the open field were calculated using DeepLabCut (version 2.3.5; [28]) for body pose estimation and SimBA (version 1.3; [29]) for calculating the total distance based on the body’s center point. Rearing behavior was manually counted using Boris software (version 7.9.7; [30]). During the open field test, the rats underwent simultaneous electrophysiological recordings.

### Electrophysiological recordings

We recorded local field potentials (LFP) in the mPFC during the rats’ resting state in the home cage, exploratory behavior in an open field arena, and under urethane anesthesia. Before the start of electrophysio-logical recordings, the rats were habituated to the head stage connection and free movement with the connected cable for at least 5 min in their home cage over a period of 5 days. Two days before the experiment, the animals were housed individually.

**Fig. 2:**
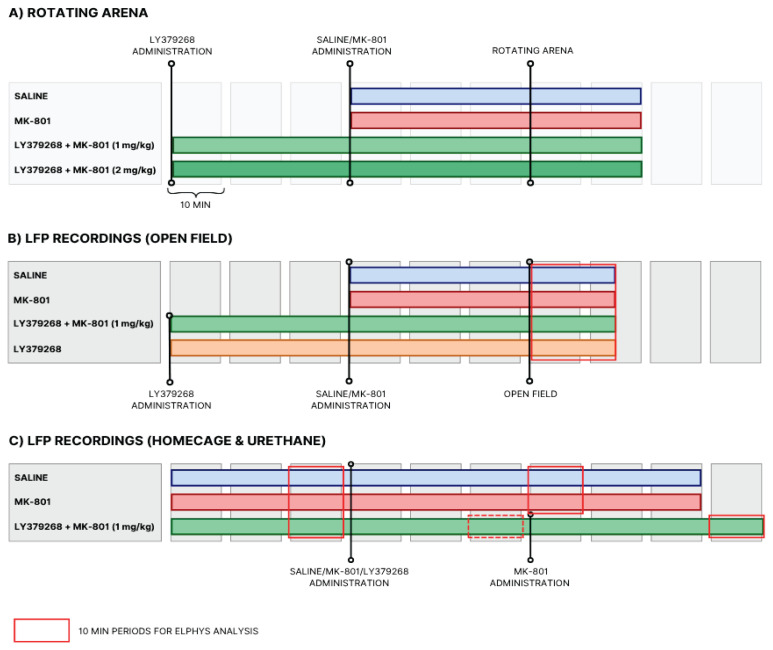
Experimental Design Overview. The experiments comprised two main groups of animals: (**A**) animals tested in the rotating arena and (**B–C**) animals used for local field potential (LFP) recordings (**A**) Rotating Arena: Animals underwent active place avoidance training on a rotating arena. LY379268 was administered intraperitoneally at doses of 1 mg/kg or 2 mg/kg, 1 h prior to each session. MK-801 (0.1 mg/kg) or saline was administered 30 min before the session. (**B**) LFP Recordings – Home Cage and Urethane Anesthesia: For electrophysiological recordings, animals were first recorded for 30 min to establish a baseline, either in the home cage or under urethane anesthesia. Subsequently, they received an injection of saline, MK-801 (0.1 mg/kg), or LY379268 (1 mg/kg). Following saline or MK-801 administration, recordings continued for 50 min. For LY379268, an additional 30-minute recording was performed prior to MK-801 administration, followed by a 50-minute post-injection recording. Red-bordered sections indicate data used for main analyses; dashed borders indicate additional segments included in the supplementary materials. Results of home cage recordings are presented in the supplementary data (Suppl. Fig. 5). (**C**) LFP Recordings – Open Field Test: The same animals from part B were also tested in the open field under simultaneous behavioral and electrophysiological recording. The injection schedule matched that of the rotating arena: LY379268 (1 mg/kg) was administered 1 h before the session, and MK-801 or saline was administered 30 min prior to the session. The open-field session lasted 15 min.

The experiment spanned three days. On the first day, the rats were recorded for two hours resting in their home cages. A 30-minute baseline was recorded before drug administration. We analyzed only the last 10 min of this period to avoid the effects of manipulation stress and to ensure the resting state of the animals. After the baseline recording, the animals received their respective treatments. For the LY379268 groups, LY379268 was injected first, followed by a 30-minute recording period, after which MK-801 was administered, and the animals were recorded for an additional 50 min. For the saline and MK-801-alone groups, the animals were injected with their respective treatments and then recorded for 50 min. On the second day, the rats were recorded only during the 15-minute open field test, and on the third day, they were recorded under anesthesia. For the anesthesia recordings, animals were injected with urethane (1000 mg/kg diluted in saline, i.p., Sigma-Aldrich) administered in three doses, each separated by 5 min, to avoid changes in blood pressure from large-volume intraperitoneal injections. Urethane anesthesia provides a stable and long-lasting anesthetic state that preserves spontaneous oscillatory brain activity and minimizes interference from behavioral states, making it suitable for assessing intrinsic network dynamics. Once the animals were fully anesthetized, we recorded a 30-minute baseline followed by a recording session with drug administration following the same injection scheme as on the first day (Fig. 2). In the supplementary data, we show the effect of LY379268 alone within the LY379268 and MK-801 group (Suppl. Fig. 3) because we did not have a separate LY379268-only group for electrophysiological recordings. This was due to the exclusion of animals with inaccurate placement of recording electrodes. By focusing on the 20–30-minute period following LY379268 injection, we tested whether LY379268 alone has an effect on brain oscillations. After the completion of all electrophysiological recordings, the animals were overdosed with isoflurane and transcardially perfused.

During the recording procedures, data from all electrodes were amplified (1000×) using Lynx-8 amplifiers (Neuralynx, USA). The signal was band-pass filtered at 1–475 Hz. A Micro 1401-3 system (CED, UK) was used to record the neural activity at a sampling rate of 2000 Hz, using Spike2 software (version 7.20) from the same company.

### Electrode placement verification

Rats from the open field test and electrophysiological experiments were perfused transcardially with 0.1 M phosphate buffer (PB), then 4 % paraformaldehyde (PFA) solution. The brains were carefully removed and post-fixed in 4 % PFA overnight, then placed in a 30 % sucrose solution with 0.1 M PB until they sank. Afterward, they were deep-frozen in dry ice. The brains were cut into 40 μm coronal slices using a Leica cryostat and stored at −20 °C for further processing. Every fourth slice was collected and processed with standard Nissl staining to verify the placement of recording electrodes. The exact location was verified according to the rat brain atlas [31].

### Data analysis and statistics

All statistical analyses were performed using GraphPad Prism (version 9.0). Electrophysiological data analyses were conducted in Matlab (R2024a, Mathworks) using custom-made scripts. All values are expressed as mean ± standard error of the mean (SEM), and statistical significance was set at p<0.05. * indicates p≤0.05, ** indicates p≤0.01, and *** indicates p≤0.001.

### Behavioral tests

Normal data distribution was tested using the Shapiro-Wilk test. Non-normal data were analyzed with the Kruskal-Wallis test and Dunn’s multiple comparison test when significant (distance moved in the open field arena and distance moved in the last five minutes). Otherwise, a one-way ANOVA with Tukey’s multiple comparison test, when significant, was used for other evaluated open field parameters (rearing behavior and time in the center of the open field arena). Regarding the rotating arena experiments, the distance moved in the arena, the number of entrances, and the number of shocks received were analyzed. A repeated-measures two-way ANOVA or mixed-effect analysis was performed with Tukey’s multiple comparisons in case of significance.

#### Electrophysiology

**Fig. 3:**
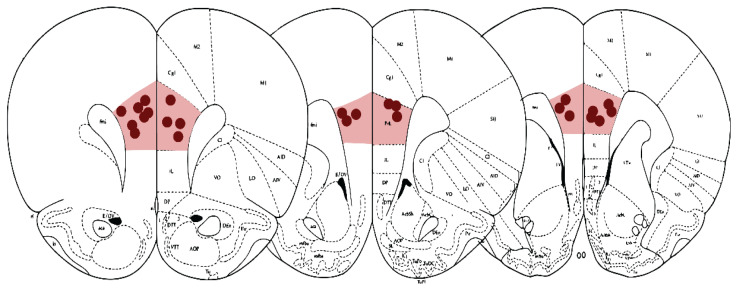
The recording electrode placement. The placement of the electrode tips in the mPFC was verified. Both electrodes were correctly placed bilaterally in eight animals, and the results from these animals were averaged. Otherwise, nine rats had a correct unilateral placement. The electrode placement locations were adapted from Paxinos and Watson [36].

For each animal subjected to electrophysiological experiments, we selected either a single electrode with accurate placement if the other was misplaced in the mPFC, or both electrodes if they were accurately placed bilaterally. In the latter case, the average value of the analyzed metrics from the two electrodes was used for subsequent data analysis. In the group receiving only LY379268, only three animals had correctly placed electrodes; due to the insufficient number of valid recordings, this group was excluded from the electrophysiological analyses. Thus, the final electrophysiological sample comprised saline (n=7), MK-801 (n=5), and MK-801 + LY379268 (n=5) animals. The locations of the electrodes used for analyzing the electrophysiological data from the included rats are illustrated in Fig. 3.

We compared the power spectral density (PSD) in low and high gamma frequency bands. In all groups, a 10-min pre-injection baseline (0–10 min) was recorded for each animal, and PSD values during the post-injection periods were expressed as a percentage of that animal’s own baseline and used for statistical comparisons. For the saline and MK-801 groups, we analyzed 30–40 min after injection. For the LY379268+MK-801 group, we analyzed 60–70 min after the LY379268 injection (corresponding to 30–40 min after the MK-801 injection). For a detailed timeline, see Section 2.5 and Fig. 2. We pre-processed the neural data for all electrophysiological analyses and used a biquad filter to filter the 50 Hz component (power line noise) and its higher harmonic frequencies. The following steps were used to calculate the average PSD in each frequency band of interest (low gamma: 30–55 Hz, and high gamma: 55–100 Hz). The LFP signal was divided into 50 % overlapping segments. An absolute Fourier spectrum was calculated for each segment and then averaged to estimate the PSD. The individual segments were multiplied by the Hanning window while estimating the PSD. Finally, the average PSD for the given frequency bands was calculated as the mean in these specific frequency bands. The analyzed intervals were 10 min long. A one-way ANOVA was used to compare the power in the low gamma (30–55 Hz) and high gamma (55–100 Hz) frequency ranges. We also computed time-frequency spectrograms between 30 and 100 Hz using a Hamming window with a size of 1 s and an overlap of 75 %.

To assess whether information processing in the mPFC was disrupted by MK-801 administration and potentially mitigated by co-application with LY379268, we analyzed the phase-amplitude coupling (PAC) between the phase of theta oscillations and the amplitude of low and high gamma oscillations using the Phase Locking Value (PLV). We calculated the PLV following Mormann *et al.* [32] and Vanhatalo *et al.* [33]. Raw data were band-pass filtered to isolate the frequencies of interest theta (4–10 Hz), low gamma (30–55 Hz), and high gamma (55–100 Hz). The filtered signals were transformed into complex analytic signals to extract phase and amplitude. For PLV, the phase was taken from the low-frequency signal and the amplitude from the high-frequency signal, with the latter Hilbert transformed to extract its phase. Phase differences between the two signals were computed, and a constant phase lag indicated coupling. The coupling strength was quantified as the length of the mean vector from averaged phase differences.

During the open field test, animals were simultaneously recorded for behavioral data using a camera and for neural activity through electrophysiological measurements. We analyzed the PSD changes (percent difference from baseline) using a one-way ANOVA for low gamma frequencies. Due to non-normal data distribution (Shapiro-Wilk test), the Kruskal-Wallis test with Dunn’s *post hoc* comparisons was used for high gamma PSD analysis. PLV for theta and low gamma, as well as for theta and high gamma, were analyzed using ordinary one-way ANOVA with Tukey’s multiple comparisons.

Urethane anesthesia was used to compare PSD and PLV after drug applications under stable conditions that eliminate the effect of behavior on brain oscillations. The animals were anesthetized, and recordings began at least 1.5 h after the final injection to ensure complete drug dissociation. We recorded 30 min of baseline activity, followed by an injection of either saline, MK-801, or pretreatment with LY379268 followed by MK-801. Each animal received the same substance as in previous tests. The LFP post-application (20–30 min after application) was compared to baseline activity using one-way ANOVA with Tukey’s *post hoc* test for theta and low gamma ranges and the Kruskal-Wallis test with Dunn’s multiple comparisons for high gamma (failed in the Shapiro-Wilk test for normality). For PLV between theta and low gamma, we used one-way ANOVA with Tukey’s comparisons. The data did not meet normality for PLV between theta and high gamma, thus necessitating the use of the Kruskal-Wallis test with Dunn’s comparisons.

## Results

### Rotating arena results

The first cohort of animals was used for the rotating arena test. The rotating arena test spanned six days, starting with one habituation day, followed by five training days in four runs. The rats received specific treatments and were trained to avoid a designated sector in the arena. Four groups were analyzed: a control group, a group treated with MK-801 alone, and two groups pretreated with LY379268 at either 1 mg/kg or 2 mg/kg, followed by MK-801 administration (Fig. 2A).

**Fig. 4:**
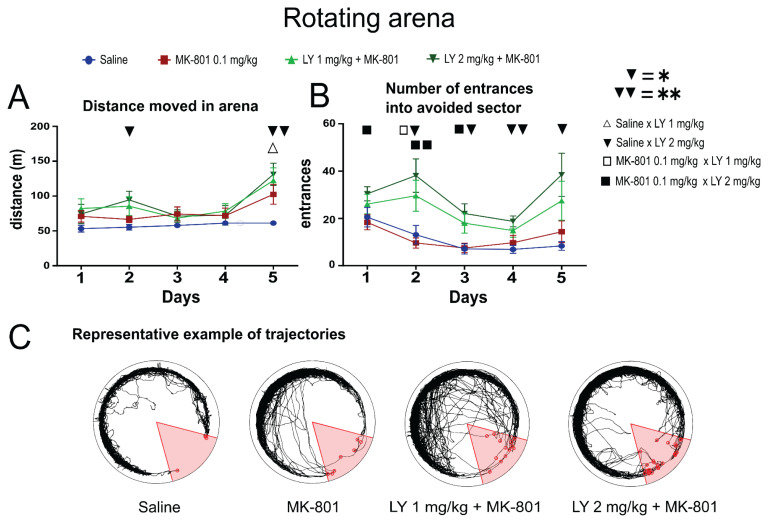
LY379268 combined with MK-801 increased locomotion and impaired avoidance learning. (**A**) Total distance moved across the five days of learning sessions. The analysis revealed a trend toward increased locomotion in MK-801-treated animals, which was further amplified in the groups receiving a combination of LY379268 (1 mg/kg or 2 mg/kg) and MK-801. This suggests that LY379268 may potentiate the hyperlocomotor effects of MK-801. Group differences are indicated as follows: difference between saline and 1 mg/kg LY379268 (△), saline and 2 mg/kg LY379268 (▼), MK-801 and 1 mg/kg LY379268 (□), and MK-801 and 2 mg/kg LY379268 (■). The level of significance is indicated by the number of symbols: one symbol represents p≤0.05, two symbols represent p≤0.01, and three symbols represent p≤0.001. (**B**) Number of entries into the avoided sector across training days. The LY379268 (2 mg/kg) + MK-801 group consistently showed higher sector entries compared to both the saline and MK-801 groups, indicating an impairment in avoidance learning. The same symbols (△, ▼, □, ■) represent significant group comparisons, as described in panel A. (**C**) Representative trajectories from each group on the final day of training (day 5), illustrating typical movement patterns in the rotating arena.

Animals changed their locomotor activity during the training (Fig. 4A). ANOVA found a significant effect of days [F_(3.207,161.2_)=7.802, p<0.001], and a main effect of groups [F_(3,51)_=3.807, p=0.0154]. As revealed by Sidak’s *post hoc* test, on the second day, there was a notable difference in the distance moved between the control group and the group pretreated with LY379268 at 2 mg/kg + MK-801 (* p=0.0336) (Fig. 4A). On the final day of training, the control group exhibited significantly lower locomotor activity compared to the groups treated with MK-801 combined with either 1 mg/kg LY379268 (* p=0.0259) or 2 mg/kg LY379268 (** p=0.0024). Significant differences were observed in the number of sector entries (effect of days: [F_(2.261,114.7_)=6.000, p=0.0023]; effect of groups: [F_(3,51)_=10.16; p<0.0001]. On the first day, a difference was observed between the MK-801-only group and the group pretreated with LY379268 (2 mg/kg) and MK-801 (* p=0.0445). By the second day, the MK-801 group pretreated with 2 mg/kg LY379268 showed a significant increase in sector entries compared to both saline controls (* p=0.0236) and the MK-801 group pretreated with 1 mg/kg LY379268 (* p=0.0492). Additionally, this group differed significantly from the MK-801-only group (** p=0.0053). On the third day of training, significant differences persisted in the number of entrances to the avoided sector, with the LY379268 (2 mg/kg) + MK-801 group showing higher entries compared to controls (* p=0.0121) and the MK-801 group (* p=0.0129). By the fourth day, only the LY379268 (2 mg/kg) + MK-801 group showed significant differences compared to controls (** p=0.0015). On the fifth and final day, a significance level (* p=0.0222) was reached between the control group and the LY379268 (2 mg/kg) + MK-801 group (Fig. 4B). To assess whether locomotor activity and task performance changed dynamically during the session due to the pharmacological effects of the drugs, each training day was divided into 5-minute intervals. This analysis, however, did not reveal any specific within-session effects in any group (Suppl. Fig. 1).

In summary, pretreatment with the mGlu2/3 receptor agonist LY379268 prior to MK-801 administration led to increased locomotion, which negatively affected task performance. This was reflected by a higher number of entries into the avoided sector during training. Notably, the saline-treated group did not differ from the MK-801 group, suggesting that the 0.1 mg/kg dose of MK-801 used in this study may not have been sufficient to induce significant locomotor or learning deficits on its own.

Additionally, variability in individual performance was observed across training days. These fluctuations may have been influenced by factors such as food intake, which could affect both behavioral outcomes and drug pharmacokinetics, as well as potential inconsistencies in the exact volume or absorption of intraperitoneally administered doses.

### Open field test results

The second cohort of animals was used for open field test and electrophysiological recordings. We used the open field test to assess the effect of the drugs on spontaneous exploration and movement. The group in the open field with LY379268 administration alone was not included in the electrophysiological data analysis due to the low number of animals with accurate electrode placement. The evaluated groups were: saline, MK-801, LY379268 1 mg/kg + MK-801 (Fig. 2B).

**Fig. 5:**
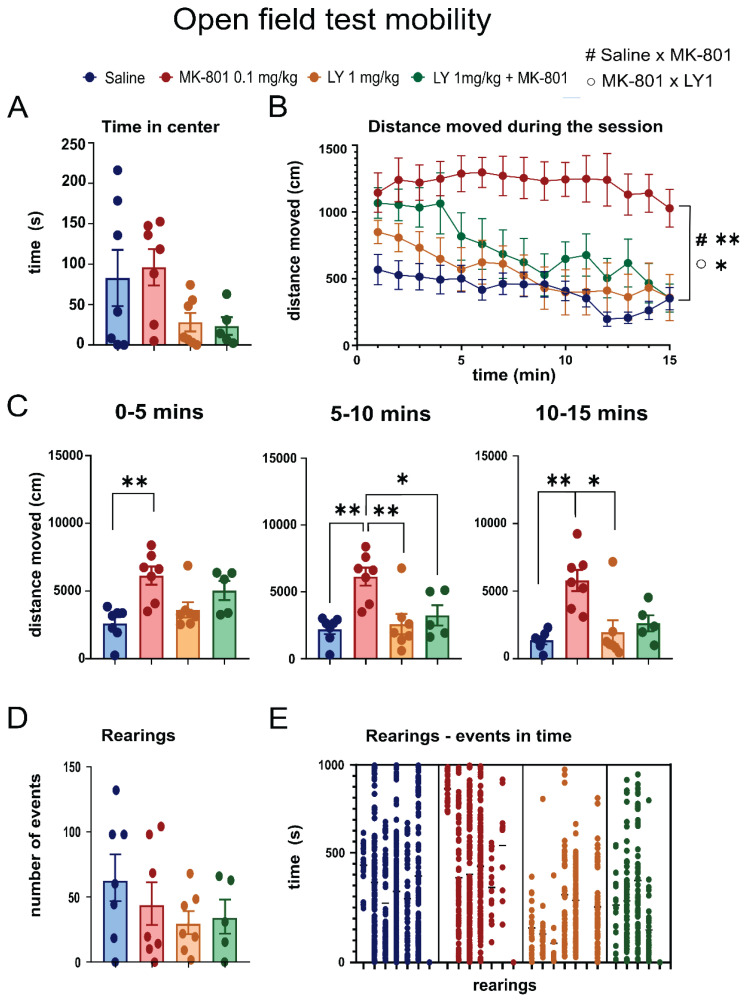
In the open field test, MK-801 increased locomotion without affecting rearing, while other groups showed normal habituation. (**A**) The time spent in the center of the open field. No significant differences were observed between groups, suggesting that neither MK-801 nor LY379268 induced anxiety-related effects during the test. (**B**) The distance moved during the last five minutes and across the 15-minute session in 1-minute bins. MK-801-treated animals maintained consistent movement throughout the session, indicating a lack of habituation. In contrast, the saline group, LY379268 group, and the LY379268 + MK-801 group exhibited a decrease in movement over time, reflecting normal habituation to the environment. The MK-801 group demonstrated a significantly greater distance moved compared to the saline group (#) and LY379268 group (○), confirming the hyperlocomotor effect of MK-801. (**C**) The total distance moved for 5 min bins. (**D**) The total number of rearings. No significant differences were observed between groups, indicating that neither MK-801 nor LY379268 affected rearing behavior. This suggests that active exploration remained unaffected despite changes in locomotion. (**E**) Rearings distribution across the 15-minute session.

The Kruskal-Wallis test showed a significant difference between groups in total distance moved (H_(3)_=12.83; ** p=0.005). The distance moved was significantly longer for the MK-801 group than for controls (* p=0.0088) or LY379268 group (* adjusted p=0.0179). When data were divided into 5-minute bins (Fig. 5C), a significant difference was observed in the first 5 min using the Kruskal-Wallis test (H_(3)_=12.55; ** p=0.0057), with *post hoc* analysis revealing a significant increase in locomotion in the MK-801 group compared to the saline group (** p=0.0061). In the second 5-minute bin (5–10 min), ordinary one-way ANOVA showed a highly significant effect (F_(3,22)_=8.825; *** p=0.0005). *Post hoc* comparisons revealed hyperlocomotion in the MK-801 group compared to all other groups (saline: ** p=0.0012; LY379268: ** p=0.0029; LY379268 + MK-801: * p=0.0272). Hyperlocomotion persisted in the last 5 min (10–15) (H_(3)_=12.85; ** p=0.0050); the MK-801 group traveled a significantly longer distance compared to controls (** p=0.0100) and the LY379268-treated group (* p=0.0142). This indicates that MK-801 induced a hyperlocomotor effect throughout the whole testing session.

Distance moved across 1-min bins in the open field was analyzed using a two-way repeated-measures ANOVA (Group × Time; Group: F_(3,22)_=8.79, p=0.0005; Group × Time: F_(13.06,95.76)_=2.32, p=0.010), and the corresponding time course shown in Figure 5B illustrates the lack of habituation in the MK-801-treated group. Results of the *post* test are in supplementary data (Table S1).

No significant difference between groups was found in the time spent in the center of the open field by ordinary one-way ANOVA (p=0.0917), suggesting no effect on anxiety-related behavior (Fig. 5A).

We further counted the number of stand-ups as a measure of rearing behavior to investigate active exploration (Fig. 5D). No significant difference was observed between the groups during the entire session (ordinary one-way ANOVA, p=0.3817). This suggests that while locomotion is increased, the ability for spontaneous exploration of a novel environment remains unchanged.

### Electrophysiological results

#### Open field test

**Fig. 6:**
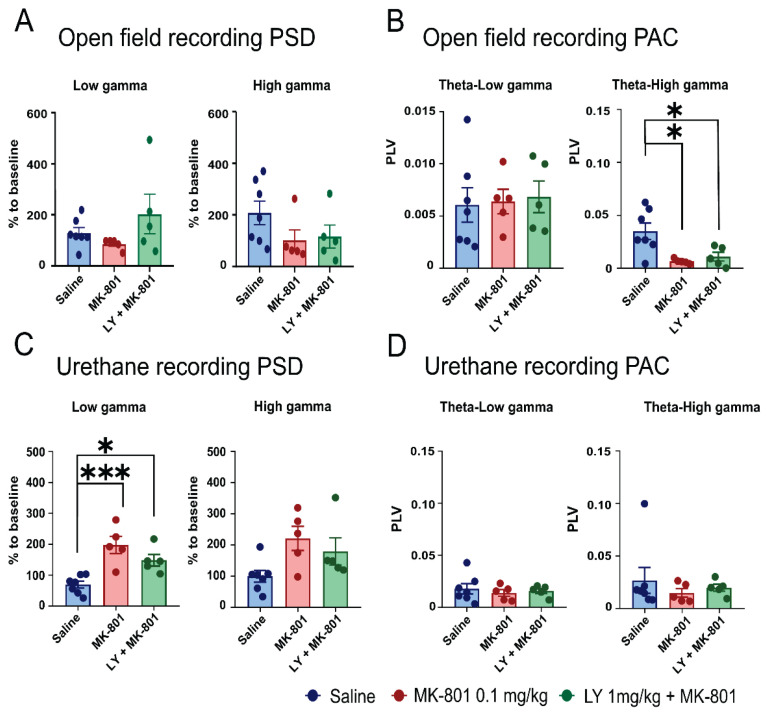
In the open field test, PSD remained unchanged across groups, but MK-801 and LY379268 + MK-801 reduced theta-high gamma PAC. (**A**) Percentual changes of PSD in low gamma (left) and high gamma (right) ranges, compared to baseline in the open field. No significant differences were observed between the groups, indicating that PSD was not significantly modulated by the treatments. (**B**) PAC is represented by phase locking value (PLV) for theta-low gamma (left) and theta-high gamma (right). No significant differences were observed in theta-low gamma coupling between groups; however, theta-high gamma PAC was significantly decreased in the MK-801 group (p=0.0120) and the LY379268 + MK-801 group (p=0.0324) compared to the saline group. LY379268 did not modulate MK-801-induced decrease in theta-high gamma PAC, as confirmed by no observed difference between the MK-801 and LY379268 + MK-801 groups. Under urethane anesthesia, both treatments increased low gamma power, while PAC remained unaffected. (**C**) Low and high gamma PSD compared to baseline. Low gamma was significantly higher in MK-801 and LY379268 + MK-801 groups compared to the saline group. (**D**) No observable differences were detected in PAC either between theta and low gamma or high gamma, as indicated by phase-locking value (PLV). Data are shown as mean ± SEM; saline n=7, MK-801 n=5, MK-801 + LY379268 n=5.

Electrophysiological analysis during the open field test, which assesses brain oscillations during active exploration, revealed selective effects of MK-801 and the combination of MK-801 with LY379268 on phase-amplitude coupling (PAC) between theta and gamma rhythms. Measuring PAC in this context was crucial as it reflects neural network coordination during spontaneous exploration. This approach allowed us to evaluate whether LY379268 could mitigate MK-801-induced deficits in neural synchronization. For the analysis of low gamma power spectral density (PSD), ordinary one-way ANOVA revealed no significant differences among the control, MK-801-treated, and LY379268 (1 mg/kg) + MK-801 groups (F_(2,14)_=1.776; p=0.2055). Similarly, the Kruskal-Wallis test for high gamma PSD showed no significant differences between groups (H_(2)_=9.394; p=0.1023) (Fig. 6A). Phase-locking value (PLV) in the theta-low gamma range, assessed using ordinary one-way ANOVA, also showed no significant differences across groups (F_(2,14)_=0.06673; p=0.9357). However, theta-high gamma PLV revealed significant group differences *via* ordinary one-way ANOVA (F_(2,14)_=6.935; ** p=0.0081). *Post hoc* analysis indicated significant reductions in theta-high gamma PLV in MK-801-treated animals compared to controls, with saline vs. MK-801 (* p=0.0120) and saline vs. LY379268 + MK-801 (* p=0.0324) both showing significant differences. No significant difference was found between the MK-801 and LY379268 + MK-801 groups. These results suggest that MK-801 reduces theta-high gamma PAC, while LY379268 does not further modulate this effect (Fig. 6B). PSD and theta-low gamma PAC remained unaffected by the treatments. We have not observed any correlation between the movement in the open field and the neuronal activity (Suppl. Fig. 4).

#### Urethane recording

Under urethane anesthesia, we assessed PSD and PAC to examine oscillatory activity in a stable, behavior-free state. Animals were anesthetized with three injections of urethane and recordings began at least 1.5 h after the final injection, ensuring full dissociation, as confirmed by the absence of responses to painful stimuli. A 30-minute baseline was recorded, followed by the administration of saline, MK-801, or a combination of MK-801 + LY379268 (Fig. 2C). Each animal received the same treatment as in previous tests. To account for the progressive effects of sedation and deepening anesthesia, PSD data from a 10-minute interval (20–30 min post-application) were compared to a 10-minute interval from the baseline recording (20–30 min).

One-way ANOVA of percentual differences in low gamma PSD (30–55 Hz) revealed significant differences between the control group and the MK-801 group (** p=0.0006) and between the control group and the MK-801 + LY379268 group (* p=0.0225; overall F_(2,14)_=12.84; *** p=0.0007) (Fig. 6C). For high gamma PSD percentage differences, the Kruskal-Wallis test showed a significant difference between groups (H_(2)_=6.579; * p=0.0290), but the *post hoc* test did not reveal a specific significance in comparisons of the groups. Ordinary one-way ANOVA for PAC between theta and low gamma (F_(2,14)_=0.2428; p=0.7877) and the Kruskal-Wallis test for theta-high gamma PAC (H_(2)_=1.042; p=0.6155) showed no significant group differences, as indicated by PLV (Fig. 6D). These results highlight the significant effects of MK-801 and its combination with LY379268 on low gamma PSD but no detectable impact on theta-gamma PAC under anesthesia. We did not observe a difference between the Saline injection and single LY379268 administration, preceding the MK-801 administration (Suppl. Fig. 3).

Furthermore, urethane recordings showed no differences in PAC between the control Saline group, the MK-801 group, and the single administration of LY379268 prior to MK-801. However, the PSD in both low and high gamma bands was lower in the LY379268 prior to the MK-801 group compared to the MK-801 group but did not differ from the Saline group (Suppl. Fig. 3). No differences were observed during electrophysiological recordings in the home cage condition (Suppl. Fig. 5).

## Discussion

Our findings reveal task-dependent effects of LY379268 on both behavior and neural activity, challenging the initial hypothesis that this mGlu2/3 receptor agonist could counteract MK-801-induced hyperlocomotion and cognitive deficits. Behaviorally, although MK-801 alone did not induce hyperlocomotion in the context of aversive learning during the rotating arena task, co-administration with LY379268, particularly at higher doses, unexpectedly resulted in pronounced hyperlocomotion. Importantly, avoidance learning itself was not significantly impaired by MK-801 alone, so the rotating arena data do not provide a direct test of whether LY379268 can restore MK-801-induced navigational deficits; instead, they highlight how LY379268 alters behavior in a context where MK-801’s effects are relatively subtle. In contrast, in a non-aversive setting – the open field test, LY379268 showed a slight reduction in hyperlocomotion when combined with MK-801, though this effect was limited to the lower dose. During exploratory behavior in the open-field test, while gamma PSD remained unchanged, there was a notable decrease in theta-high gamma PAC after both MK-801 or MK-801 and LY379268 coapplication, indicating disrupted network synchronization. Under urethane anesthesia, we observed increased low gamma power following MK-801 administration, which LY379268 failed to mitigate.

### Behavioral responses across contexts

Our findings indicate that the mGlu2/3 receptor agonist LY379268 exerts nuanced, task-dependent effects in the MK-801 model, and that these effects must be interpreted in light of how strongly the model itself is expressed in each paradigm. In the non-aversive open field, the 0.1 mg/kg dose of MK-801 produced the expected robust hyperlocomotion and reduced habituation, consistent with previous reports using similar doses. In contrast, in the rotating arena the same dose, chosen following Svoboda *et al.* [27], did not reliably increase locomotion or impair avoidance learning; saline- and MK-801-treated rats performed similarly across days, in line with Kubík *et al.* [34], who also reported that this dose may be insufficient to induce elevated locomotor activity in this task. Consequently, in this aversive learning context there was no clear MK-801-induced cognitive deficit that LY379268 could mitigate. Instead, LY379268 modulated behavior on top of largely preserved baseline performance: when co-administered with MK-801 during avoidance learning with footshocks, it paradoxically intensified hyperlocomotion and, at 2 mg/kg, impaired avoidance behavior that was not detectably affected by MK-801 alone. This pattern contrasts with earlier reports that mGlu2/3 agonists reduce NMDA antagonist-induced hyperactivity in simpler, less aversive paradigms [23,35–37], and with Sokolenko *et al.* [38], where similar treatments ameliorated impaired working memory. Together, these discrepancies suggest that LY379268’s behavioral effects are not uniformly cognition-enhancing but depend critically on task demands, motivational state, and the specific conditions under which NMDA receptor hypofunction is induced. The notable differences observed between our results from the rotating arena and the open field test may be attributed to the renewal of the breeding colony at the institutional animal facility. Bueno *et al.* [39] demonstrated that the effects of MK-801 can vary across different rat strains. Thus, the two cohorts of animals used in our experiments – one for the rotating arena and the other for the open field – could differ genetically, potentially influencing their sensitivity to MK-801.

### Dose-dependency and E/I balance in non-aversive contexts

The influence of LY379268 in reducing MK-801-induced hyperlocomotion in the open field test supports the hypothesis that this mGlu2/3 agonist can restore E/I balance under conditions where pathological excitatory drive is less pronounced [20,40]. In our study, LY379268, administered at a dose of 1 mg/kg alone, did not significantly affect spontaneous locomotion. Importantly, when co-administered with MK-801, it attenuated the hyperlocomotion otherwise induced by MK-801, suggesting a potential regulatory effect on glutamate-driven hyperexcitability. However, this modulatory effect was not observed in the more demanding rotating arena task, where the same combination resulted in increased locomotion and impaired performance. These findings suggest that the 1 mg/kg dose of LY379268 may represent an intermediate condition – subthreshold on its own but capable of modulating excessive excitation under certain conditions. This is in line with previous work showing that lower doses of LY379268 have limited behavioral impact [41], while higher doses (more than 3 mg/kg) are required to fully reverse NMDA antagonist-induced disruptions [41,42]. The divergence in behavioral outcomes across paradigms further emphasizes the role of environmental and task-related factors in shaping the functional effects of glutamatergic modulation.

### Neural correlates: LFP and oscillatory dynamics in varying contexts

To better understand the mechanistic underpinnings of these behavioral outcomes, we examined LFP in the mPFC across two distinct conditions: active exploration in the open field and under urethane anesthesia. In the open field test, a mildly stressful and novel environment – neither MK-801 alone nor in combination with LY379268 altered low or high gamma power in the mPFC. Previous studies in rats demonstrated increased gamma power in the resting state following systemic MK-801 application in the mPFC [43] or frontal cortex [44]. However, both studies employed a higher dose (0.2 mg/kg) than used in our experiments. Similarly, Cui *et al.* [45] reported that prefrontal gamma oscillations remained unchanged after MK-801 administration at 0.1 mg/kg but increased significantly at 0.3 mg/kg in mice. On the contrary, Hiyoshi *et al.* [46] showed that MK-801 (0.1 mg/kg) significantly increased cortical gamma oscillation power in rats. Similarly, MK-801 at a dose of 0.08 mg/kg caused only a mild increase in gamma oscillations in cortical electroencephalography (EEG) recordings in rats during open field exploration, while a dose of 0.16 mg/kg had a much more pronounced effect [47]. The contrasting effects on gamma power in previous studies highlight potential species, region, or methodological differences. Altogether, these findings suggest task-dependent and dose-dependent effects of NMDA receptor antagonists in modulating cortical oscillations. Although our study employed a single MK-801 dose (0.1 mg/kg), comparisons with earlier literature allow us to contextualize our findings within a broader dose-response framework. Higher doses elicit robust gamma increases, while lower doses may insufficiently engage NMDA receptor-dependent circuits.

In addition, MK-801 and LY379268 + MK-801 groups exhibited reduced theta-high gamma PAC in the open field test, indicating disrupted network synchronization. This aligns with observations by Abad-Perez *et al.* [11], who reported that MK-801 at a dose of 0.075 mg/kg impairs theta-gamma PAC in the mPFC in mice. Although LY379268 partially moderated hyperlocomotion at MK-801 dose of 0.1 mg/kg in the open field test, it did not restore normal oscillatory patterns. Interestingly, LY379268 dose-dependently reversed MK-801-induced increases in cortical gamma power in rats [46]. However, in the present study, LY379268 failed to restore theta-gamma PAC, suggesting that while LY379268 can attenuate some aspects of network hyperactivity under certain conditions, it may not be sufficient to restore more complex forms of network synchrony. Disrupted network synchrony in the mPFC may reflect impaired cognitive processes, including attention and memory [48], which rely heavily on coherent theta-gamma interactions. This suggests that while LY379268 at lower doses can modestly modulate simple behaviors as locomotion, its capacity to normalize neuronal oscillations possibly involved in higher cognitive functions is limited.

Because theta-gamma PAC and gamma power can depend on locomotor state, we examined the relationship between open field movement and our electrophysiological measures. We did not observe any significant correlation between locomotion and gamma power or theta-high gamma PAC (Suppl. Fig. 3), and MK-801 also enhanced low-gamma power under urethane anesthesia, in the absence of movement. These observations make a purely behavioral explanation unlikely, although we acknowledge that our PAC analyses were not performed in speed- or state-binned epochs, which remains an important direction for future work.

### Electrophysiological effects under urethane anesthesia

Recordings under urethane anesthesia revealed that MK-801 increased low gamma power in the mPFC, confirming earlier reports that gamma hyperactivity persists even when motor and environmental influences are minimized [47]. This intrinsic cortical circuit dysfunction is characteristic of NMDA receptor antagonist challenges and implicates impairments in PV+ interneurons critical for maintaining cortical rhythmogenesis [8,49,50]. Contrary to Jones *et al.* [49], indicating that mGlu2/3 agonists can reverse ketamine-induced gamma abnormalities, LY379268 here failed to attenuate the MK-801-driven increase in low gamma power. This discrepancy may be explained by differences in antagonist pharmacodynamics – ketamine and MK-801 target NMDA receptors in distinct ways – and insufficient LY379268 dosing [42]. In addition, Hiyoshi *et al.* [46] demonstrated that even a 1 mg/kg dose of LY379268 was sufficient to begin reversing the MK-801 (0.1 mg/kg)-induced increase in cortical gamma oscillation in awake rats. Collectively, these results indicate that the efficacy of LY379268 in normalizing NMDA receptor antagonist-induced gamma abnormalities is highly task-dependent, potentially influenced by factors such as brain state, antagonist pharmacology, and dose parameters.

### Integrating findings and reconciling discrepancies

Several factors likely underlie the inconsistencies between our results and those reported elsewhere. First, dose dependency is critical. While some studies revealed that low LY379268 doses leave baseline exploration unchanged [41], other studies demonstrated that only higher doses are capable of fully restoring normal network activity or behavior disrupted by NMDA antagonists. For example, Fujáková *et al.* [42] used a dose of 30 mg/kg, which induced more pronounced behavioral deficits. Secondly, the motivation and arousal level associated with the task appear central to the effects of LY379268. Tasks that involve aversive stimuli, such as footshock, engage widespread neuromodulatory systems that may override the capacity of LY379268 to re-establish normal glutamatergic tone. Conversely, non-aversive contexts allow LY379268 to counteract MK-801-induced excitation, albeit modestly.

Finally, our electrophysiological results underscore the complexity of mGlu2/3 modulation. From the task demands to the anesthetized state, contextual factors can influence whether LY379268 alters gamma power, theta-gamma PAC, or remains electrophysiologically silent. Such context- and state-dependence may explain why LY379268, despite the preclinical promise, has not advanced into routine clinical use.

## Limitations and future directions

Our study’s limitations include the relatively narrow dose ranges of LY379268 and MK-801 examined and the use of primarily rodent models. Future work should employ a broader range of agonist and antagonist doses, explore additional behavioral paradigms (including those that vary systematically in arousal), and combine electrophysiological techniques with optogenetics or chemogenetics to isolate circuit-specific mechanisms. Moreover, investigating the interplay of mGlu2/3 modulation with other receptor systems (e.g., GABA-B, AMPA) may provide a more integrated picture of how excitatory and inhibitory circuits are balanced under pathological conditions. An additional limitation lies in our inability to form a separate LY379268 group for rotation arena learning and electrophysiology recordings due to the exclusion of animals with incorrect electrode placements and the impossibility of adding this group subsequently due to the risk of batch effects. To address this limitation, we analyzed LFP in the LY379268 + MK-801 group prior to the administration of MK-801 and found no differences between controls and LY379268 administration (Suppl. Fig. 2). Consequently, for the rotating arena and electrophysiological data we cannot definitively determine whether the observed effects reflect a specific interaction between MK-801 and LY379268 or are primarily driven by LY379268 alone; the co-administration results should therefore be interpreted as the net effect of the combined treatment. Another limitation is that in the open field test, only the 1 mg/kg dose of LY379268 was used, and inclusion of both doses could have provided deeper insight into dose-dependent effects on spontaneous behavior and cortical dynamics.

## Conclusions

In summary, our results highlight the complexity and conditionality of the actions of LY379268 on MK-801-induced behavioral and electrophysiological alterations. Rather than universally ameliorating NMDA receptor antagonist-induced disruptions, LY379268’s efficacy depends on the environmental context, the motivational and emotional state of the animal, and the dose employed. LY379268 paradoxically exacerbated hyperlocomotion in aversive learning paradigms, whereas in open field conditions, it slightly reduced hyperactivity at a low dose without fully restoring normal neural synchrony. LY379268 could not reverse the disruption of theta-high gamma PAC in the open field or the persistent gamma power increase under anesthesia. These findings underscore the intricate interplay between mGlu2/3 and NMDA receptor signaling. For future studies, including a group receiving LY379268 alone in the rotating arena would allow a more precise characterization of its isolated effects in aversive learning contexts, complementing the behavioral assessment already conducted in the open field. Ultimately, a deeper understanding of the task- and dose-dependent nature of LY379268’s effects is crucial for refining therapeutic strategies targeting glutamatergic dysregulation and guiding the translation of these findings into clinical interventions.

## Supplementary Information
