# Supplementary material for: Task-Specific Effects of mGlu2/3 Receptor Agonist LY379268 on MK-801-Induced Behavioral and Neural Dysfunctions in Rats
Source: Physiol Res. 2026 Feb 1;75(1):149–66. doi: 10.33549/physiolres.935715 (PMC13127986; doi:10.33549/physiolres.935715)
Supplement: Supplementary file 1 [file 75_149_Suppl_Fig_1.pdf]

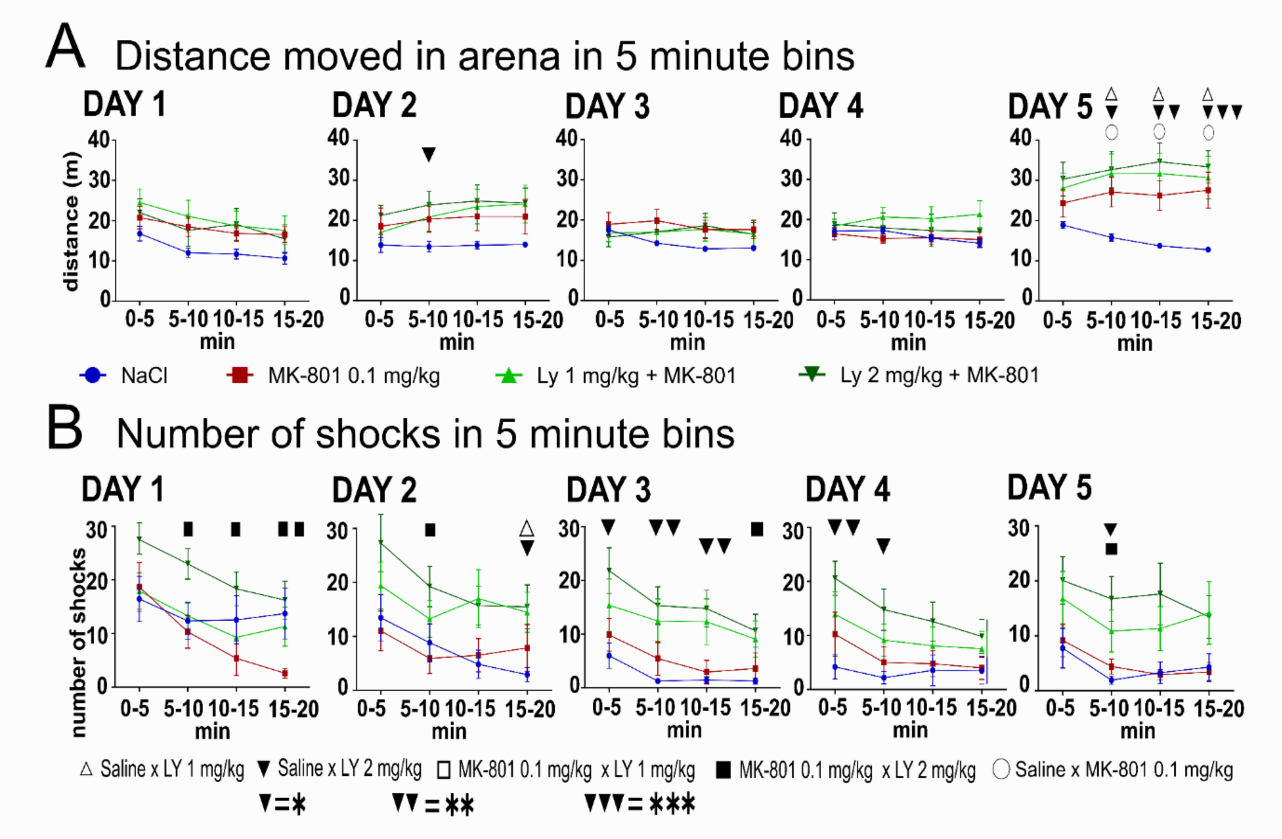

Supplementary Fig. 1. In the rotating arena, within-session analysis revealed no early differences, but significant increases in locomotor activity emerged on day 5 in MK-801 and LY379268 + MK-801 groups compared to saline: *(A) Distance moved in 5-minute intervals across the five days of learning. Group differences are indicated as follows: saline vs. 1 mg/kg LY379268 (△), saline vs. 2 mg/kg LY379268 (▼), MK-801 vs. 1 mg/kg LY379268 (□), MK-801 vs. 2 mg/kg LY379268 (■), and saline vs. MK-801 (○) The level of significance is indicated by the number of symbols: one symbol represents p≤0.05, two symbols represent p≤0.01, and three symbols represent p≤0.001.* **Within-session analysis of shocks received revealed that LY379268 (2 mg/kg) combined with MK-801 consistently led to higher shock counts across multiple days and time bins, indicating worsened avoidance behavior compared to both saline and MK-801 groups.** *(B) The number of shocks received across training days was presented in 5-minute time bins.*

To explore the dynamic effects of pharmacological treatments during each session, we divided the training periods into 5-minute bins and analyzed locomotor activity across the five days of learning (presented in 5-minute time bins; Suppl. Fig. 1A). Distance moved was analyzed for each day using a two-way repeated-measures ANOVA with Group as a between-subject factor and Time bin as a within-subject factor (Day 1: time × group, $F(9,150)=0.5561$, $p=0.8311$; time, $F(2.181,109.0)=15.39$, $p<0.0001$; group, $F(3,50)=1.040$, $p=0.3831$; Day 2: time × group, $F(9,153)=0.6151$, $p=0.7829$; time, $F(1.759,89.71)=3.234$, $p=0.0502$; group, $F(3,51)=1.582$, $p=0.2050$; Day 3: time × group, $F(9,150)=1.295$, $p=0.2440$; time, $F(1.772,88.60) = 0.8583$, $p=0.4155$; group, $F(3,50)=0.4990$, $p=0.6846$; Day 4: time × group, $F(9,147)=0.8720$, $p = 0.5518$; time, $F(2.096,102.7)=0.5231$, $p=0.6027$; group, $F(3,49)=0.5916$, $p=0.6235$; Day 5: time × group, $F(9,150)=1.482$, $p=0.1592$; time, $F(1.557,77.84)=0.5380$, $p=0.5421$; group, $F(3,50)=3.822$, $p=0.0153$). On day 1, no *post hoc* group differences were detected. On day 2, a single significant difference was observed in the 5-10 min bin between the saline and LY 2 mg/kg + MK-801 groups (*p=0.0427), with no other pairwise differences. On days 3 and 4, no significant *post hoc* group differences were found in any bin. In contrast, on day 5, robust differences emerged across the later bins: in the 5-10 min bin, the saline group differed from MK-801 (*p=0.0449), LY 1 mg/kg + MK-801 (*p=0.0297) and LY 2 mg/kg + MK-801 (*p=0.0103); in the 10-15 min bin, saline differed from MK-801 (*p=0.0238), LY 1 mg/kg + MK-801 (*p=0.0176) and LY 2 mg/kg + MK-801 (**p=0.0020); and in the 15-20 min bin, saline again differed from MK-801 (*p=0.0279), LY 1 mg/kg + MK-801 (*p=0.0252) and LY 2 mg/kg + MK-801 (***p=0.0006), indicating persistently higher locomotor activity in all MK-801–treated groups at the end of

training.

For shocks received in the avoided sector, within-session effects were analyzed using a two-way repeated-measures ANOVA with Group and Time bin (0-5, 5-10, 10-15, 15-20 min) as factors (Suppl. Fig. 1B). For day 1, the analysis showed a significant main effect of time ($F(1.766,88.30)=19.71$, $p<0.0001$) and group ($F(3,50)=3.252$, *$p=0.0293$), but no time × group interaction ($F(9,150)=1.741$, $p=0.0845$). On day 2, there were again significant main effects of time ($F(1.690,86.21)=7.643$, **$p=0.0016$) and group ($F(3,51)=3.306$, *$p=0.0274$), without a significant time × group interaction ($F(9,153)=0.9411$, $p=0.4915$). On day 3, both time ($F(1.671,85.20)=11.28$, ***$p=0.0001$) and group ($F(3,51)=4.192$, **$p=0.0100$) were significant, whereas the time × group interaction was not ($F(9,153)=0.6577$, $p=0.7460$). On day 4, the ANOVA revealed significant main effects of time ($F(2.108, 105.4)=10.61$, $p<0.0001$) and group ($F(3,50)=2.837$, *$p=0.0473$), but again no time × group interaction ($F(9,150)=1.249$, $p=0.2694$). Finally, on day 5, significant main effects of time ($F(2.043,102.2)=7.296$, **$p=0.0010$) and group ($F(3,50)=3.138$, *$p=0.0334$) were observed, with no significant time × group interaction ($F(9,150)=0.8202$, $p=0.5984$).

Several within-session *post hoc* differences were detected. On day 1, significant differences emerged between MK-801 and LY 2 mg/kg + MK-801 in the 5-10 minute bin (*$p=0.0252$), 10-15 minute bin (*$p=0.0348$), and 15-20 minute bin (**$p=0.0075$). On day 2, differences were found between saline and LY 2 mg/kg + MK-801 in the 5-10 minute bin (*$p=0.0394$), and between saline and both LY 1 mg/kg + MK-801 (*$p=0.0449$) and LY 2 mg/kg + MK-801 (*$p=0.0386$) in the 15-20 minute bin. On day 3, group differences emerged early: saline vs. LY 2 mg/kg + MK-801 in the 0-5 minute bin (*$p=0.0173$), 5-10 minute bin (**$p=0.0059$), and 10-15 minute bin (**$p=0.0072$); additionally, MK-801 vs. LY 2 mg/kg + MK-801 showed significance at 10-15 minutes (*$p=0.0354$). On day 4, only the first two bins showed significant differences between saline and LY 2 mg/kg + MK-801 (**$p=0.0014$ and *$p=0.0258$, respectively). On the final day (day 5), significant differences were again observed: MK-801 vs. LY 2 mg/kg + MK-801 in the 0-5 minute bin (*$p=0.0445$), and in the 5-10 minute bin, saline vs. LY 2 mg/kg + MK-801 (*$p=0.0236$), MK-801 vs. LY 1 mg/kg + MK-801 (*$p=0.0492$), and MK-801 vs. LY 2 mg/kg + MK-801 (**$p=0.0053$). In the 10-15 minute bin, both saline vs. LY 2 mg/kg + MK-801 (*$p=0.0121$) and MK-801 vs. LY 2 mg/kg + MK-801 (*$p=0.0129$) were significant. In the final 15-20 minute interval, a difference was observed between saline and LY 2 mg/kg + MK-801 (**$p=0.0015$).
