# Supplementary material for: Task-Specific Effects of mGlu2/3 Receptor Agonist LY379268 on MK-801-Induced Behavioral and Neural Dysfunctions in Rats
Source: Physiol Res. 2026 Feb 1;75(1):149–66. doi: 10.33549/physiolres.935715 (PMC13127986; doi:10.33549/physiolres.935715)
Supplement: Supplementary file 2 [file 75_149_Suppl_Fig_2.pdf]

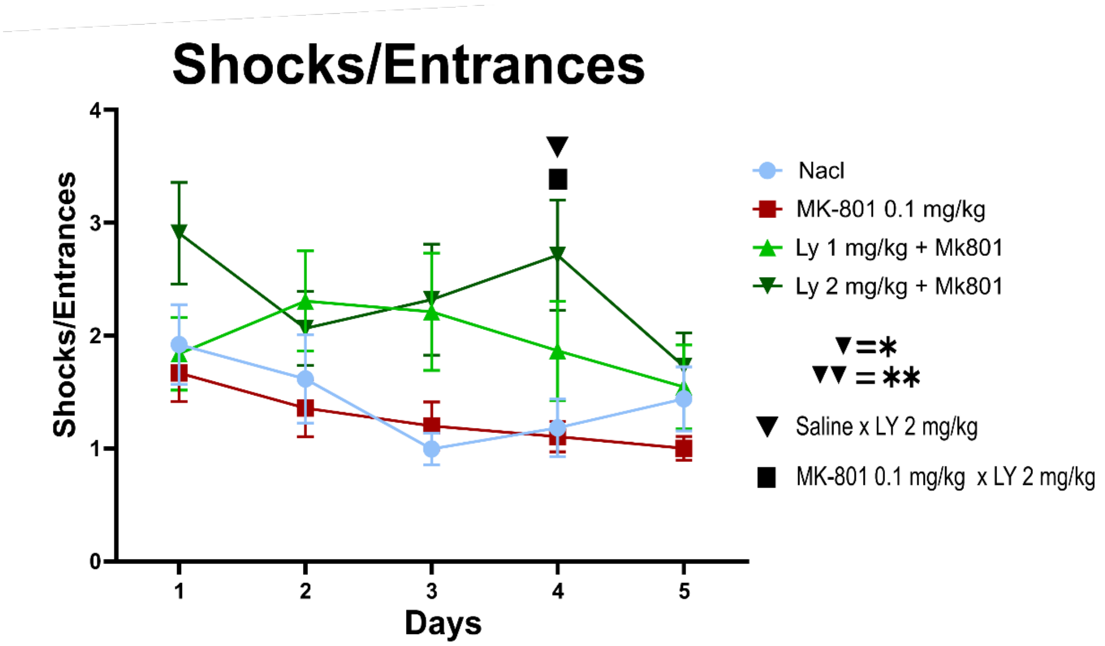

Supplementary Fig. 2. The *shocks-per-entrance* parameter revealed an effect of LY379268 (2 mg/kg) administration compared to the saline and MK-801 groups on Day 4 in the rotating arena.

To examine procedural learning in the arena, we calculated the number of shocks per entrance (formula: shocks / (entrances + 1) to avoid division by zero). A two-way ANOVA revealed an effect of days (F(3.449,175.0)=2.907, p=0.0296) and an effect of group (F(3,51)=3.709, p=0.0173), but no interaction (F(10.35,175.0)=1.275, p=0.2461). Tukey's *post hoc* test showed a significant difference between the LY379268 (2 mg/kg) and saline groups, and between the LY379268 (2 mg/kg) and MK-801 (0.1 mg/kg) groups, indicating that the slightly worsened procedural learning observed in the LY379268 (2 mg/kg) group reached statistical significance only on this particular day.
