# Supplementary material for: Task-Specific Effects of mGlu2/3 Receptor Agonist LY379268 on MK-801-Induced Behavioral and Neural Dysfunctions in Rats
Source: Physiol Res. 2026 Feb 1;75(1):149–66. doi: 10.33549/physiolres.935715 (PMC13127986; doi:10.33549/physiolres.935715)
Supplement: Supplementary file 3 [file 75_149_Suppl_Fig_3.pdf]

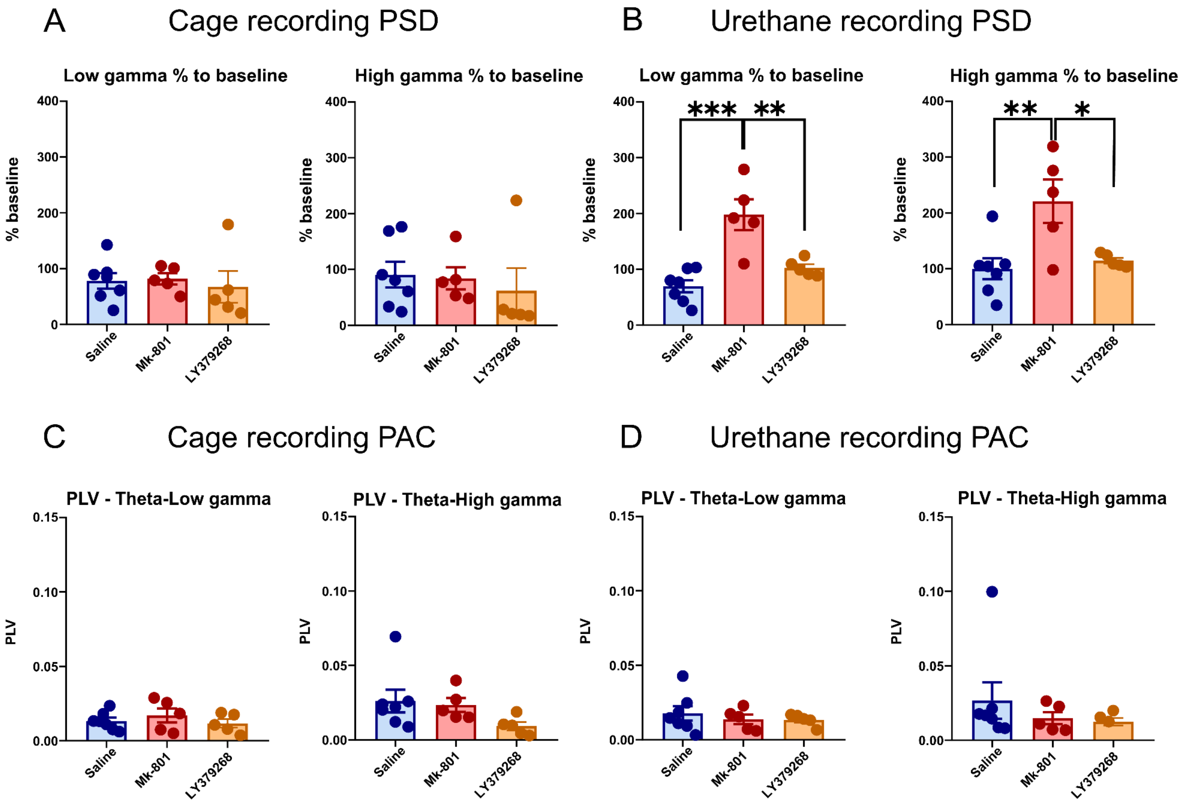

Supplementary Fig. 3. We did not observe any effect of LY379268 administration on local field potentials (LFPs) in the mPFC. *(A) No significant effect of treatment on PSD was observed in the low and high gamma ranges compared to baseline activity in home cage recordings. (B) In urethane recordings, significant differences in power spectral density (PSD) were observed between the control (saline) group and the MK-801 group, as well as between the MK-801-treated group and the LY379268 group. However, no significant differences were found between the control group and the LY379268 group in both low and high gamma ranges. (C) In home cage recordings, there were no significant differences in phase-amplitude coupling (PAC) between theta and low gamma or between theta and high gamma, as indicated by phase-locking value (PLV). (D) Similarly, in urethane recordings, no significant differences in PAC were observed between theta and low gamma or between theta and high gamma, as indicated by PLV. Data are shown as mean ± SEM; saline n=7, MK-801 n=5, MK-801 + LY379268 n=5.*

We analyzed neural activity in rats under both home cage conditions and urethane anesthesia after the administration of LY379268 but before the administration of MK-801 (see Fig. 2, marked by a dashed line) to assess whether LY379268 alone induces differences in PSD or theta-gamma PAC compared to controls.

Under home cage conditions, ordinary one-way ANOVA showed no significant differences in PSD in the low gamma range (F(2,14)=0.1496; p=0.8624), and the Kruskal-Wallis test similarly showed no significant differences in the high gamma range (H(2)=3.227; p=0.2084). In urethane recordings, however, ordinary one-way ANOVA revealed significant differences in the low gamma range (F(2,14)=16.31; ***p=0.0002), with *post hoc* tests showing significant differences between the control group and the MK-801-treated group (***p=0.0002) and between the MK-801-treated group and the LY379268-treated group (p=0.0045). For high gamma under urethane anesthesia, ordinary one-way ANOVA also indicated significant differences (F(2,14)=7.298; **p=0.0067), with *post hoc* tests identifying significant differences between the control group and the MK-801-treated group (**p=0.0081) and between the MK-801-treated group and the LY379268-treated group (*p=0.0206).

For theta-gamma PAC, in home cage recordings, ordinary one-way ANOVA showed no significant differences in PLV between theta and low gamma (F(2,14)=0.6312; p=0.5467). While the Kruskal-Wallis test displayed a significant difference in theta-high gamma PAC (H(2)=6.413; *p=0.0339), *post hoc* tests did not reveal any significant pairwise differences. In urethane conditions, ordinary one-way ANOVA showed no significant differences in PLV between theta and low gamma (F(2,14)=0.3888; p=0.6849). Similarly, the Kruskal-Wallis test showed no significant

differences in theta-high gamma PAC ($H(2)=1.250$; $p=0.5545$). These findings indicate that LY379268 alone does not induce significant changes in PSD or PAC compared to controls under either condition.
