# Supplementary material for: Task-Specific Effects of mGlu2/3 Receptor Agonist LY379268 on MK-801-Induced Behavioral and Neural Dysfunctions in Rats
Source: Physiol Res. 2026 Feb 1;75(1):149–66. doi: 10.33549/physiolres.935715 (PMC13127986; doi:10.33549/physiolres.935715)
Supplement: Supplementary file 4 [file 75_149_Suppl_Fig_4.pdf]

A Correlation of Distance moved and Power Spectral Density

Correlation: Distance moved/ Low gamma % to baseline

	Saline	MK-801	LY + MK-801
Spearman r			
r	0.3571	0	-0.5
95% confidence interval			
P value			
P (two-tailed)	0.4444	>0.9999	0.45
P value summary	ns	ns	ns
Exact or approximate P value?	Exact	Exact	Exact
Significant? (alpha = 0.05)	No	No	No

Correlation: Distance moved/ High gamma % to baseline

	Saline	MK-801	LY + MK-801
Spearman r			
r	-0.3214	0.6	-0.1
95% confidence interval			
P value			
P (two-tailed)	0.4976	0.35	0.95
P value summary	ns	ns	ns
Exact or approximate P value?	Exact	Exact	Exact
Significant? (alpha = 0.05)	No	No	No

B Correlation of Distance moved and Phase-Amplitude Coupling

Correlation: Distance moved/ PLV Theta-Low gamma

	Saline	MK-801	LY + MK-801
Pearson r			
r	-0.9234	-0.4493	-0.2854
95% confidence interval	-0.9888 to -0.5594	-0.9536 to 0.7173	-0.9328 to 0.7977
R squared	0.8527	0.2019	0.08145
P value			
P (two-tailed)	0.003	0.4478	0.6416
P value summary	**	ns	ns
Significant? (alpha = 0.05)	Yes	No	No

Correlation: Distance moved/ PLV Theta-High gamma

	Saline	MK-801	LY + MK-801
Pearson r			
r	0.2731	0.3201	0.0341
95% confidence interval	-0.6042 to 0.8511	-0.7834 to 0.9376	-0.8745 to 0.8896
R squared	0.07459	0.1024	0.001163
P value			
P (two-tailed)	0.5534	0.5995	0.9566
P value summary	ns	ns	ns
Significant? (alpha = 0.05)	No	No	No

Supplementary Fig. 4. Distance moved correlates with theta-low Gamma PAC in controls, but not with PSD in any group. *(A) No significant correlation was observed between distance moved and power spectral density (PSD) in either the low gamma or high gamma frequency ranges. (B) A significant correlation between distance moved and phase-amplitude coupling (PAC) was found only in the control group for theta-low gamma frequencies. No significant correlations were observed in the other groups or for theta-high gamma coupling.*
