# Supplementary material for: Task-Specific Effects of mGlu2/3 Receptor Agonist LY379268 on MK-801-Induced Behavioral and Neural Dysfunctions in Rats
Source: Physiol Res. 2026 Feb 1;75(1):149–66. doi: 10.33549/physiolres.935715 (PMC13127986; doi:10.33549/physiolres.935715)
Supplement: Supplementary file 5 [file 75_149_Suppl_Fig_5.pdf]

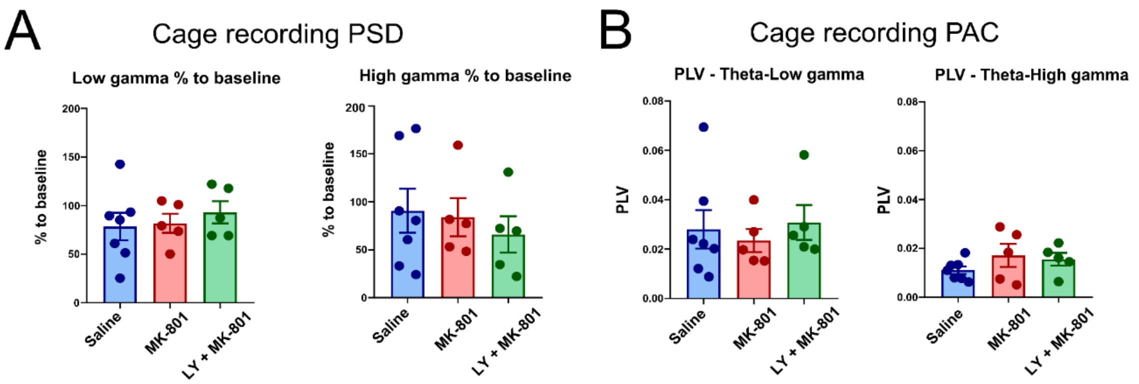

Supplementary Fig. 5. In home cage recordings, there was no effect of drug treatments on PSD and PAC: Power Spectral Density (PSD) and Phase-Amplitude Coupling (PAC) in the home cage. *(A) No significant effect of treatment on PSD was observed in both low and high gamma ranges compared to baseline activity. (B) There were no observable differences in PAC between theta and lower gamma or between theta and higher gamma, as indicated by phase-locking value (PLV). Data are shown as mean ± SEM; saline n=7, MK-801 n=5, MK-801 + LY379268 n=5.*

Home cage recording

We compared measured power to baseline activity for electrophysiological recordings in the home cage. Baseline activity was defined using a 10-minute period from 20 to 30 min after connecting the animal to the electrophysiological setup. This time frame was chosen to minimize stress-related activity from the initial head stage connection and to provide the animal with adequate time to acclimate to the setup. The baseline period was then compared to a 10-minute period recorded 30 to 40 min after the injection of saline, MK-801, or MK-801 following a prior injection of LY379268 (1 mg/kg). See Fig. 2 for a detailed experimental scheme.

Electrophysiological recordings in the home cage showed no significant differences in PSD or PLV across treatment groups. Ordinary one-way ANOVA revealed that the application of MK-801 or the combination of LY379268 (1 mg/kg) and MK-801 had no significant effect on PSD in the low gamma band ($F(2,14)=0.3557$; $p=0.7069$) or high gamma band ($F(2,14)=0.3429$; $p=0.7155$) as shown in Fig. 5A. Similarly, no significant differences were observed in PLV for theta-low gamma PAC ($F(2,14)=0.3750$; $p=0.694$) or theta-high gamma PAC ($F(2,14)=0.2486$; $p=0.7833$) (Fig. 5C). These findings indicate that, under home cage conditions, neither MK-801 nor the combination of LY379268 + MK-801 significantly altered the neural activity in the mPFC, suggesting that the treatments did not disrupt baseline oscillatory dynamics or PAC in this setting.

In addition, no differences were observed in PSD or PLV between the control saline, the MK-801 group, and the single administration of LY379268, which preceded MK-801 administration, in home cage recordings (Suppl. Fig. 5).
