# Supplementary material for: Task-Specific Effects of mGlu2/3 Receptor Agonist LY379268 on MK-801-Induced Behavioral and Neural Dysfunctions in Rats
Source: Physiol Res. 2026 Feb 1;75(1):149–66. doi: 10.33549/physiolres.935715 (PMC13127986; doi:10.33549/physiolres.935715)
Supplement: Supplementary file 6 [file 75_149_Suppl_Table_1.pdf]

Supplementary Table 1. Results of the *post hoc* tests from Two-way repeated measures ANOVA for distance moved in Open field by one minute bins.

1 minute bins - distance in Open field						
Tukey's multiple comparisons test						
Time bin	Treatements	Mean diff.	95.00% CI of diff.	Adjusted P	Summary
0-1 min	Saline vs. MK-801	-576.5	-1135 to -18.41	0.0423	*
	Saline vs. LY379268 and MK801	-499.3	-995.9 to -2.706	0.0487	*
1-2 min					
	Saline vs. MK-801	-714.2	-1309 to -119.8	0.0185	*
	Saline vs. LY379268 and MK801	-526.4	-1023 to -29.73	0.0377	*
2-3 min					
	Saline vs. MK-801	-705.9	-1202 to -209.5	0.006	**
3-4 min					
	Saline vs. MK-801	-756.5	-1257 to -255.9	0.0037	**
4-5 min					
	Saline vs. MK-801	-787	-1292 to -281.9	0.0031	**
	MK-801 vs. LY379268	717.3	88.64 to 1346	0.0243	*
5-6 min					
	Saline vs. MK-801	-879.8	-1293 to -466.7	0.0003	***
	MK-801 vs. LY379268	673.7	69.98 to 1277	0.0281	*
6-7 min					
	Saline vs. MK-801	-808.6	-1334 to -283.7	0.0042	**
	MK-801 vs. LY379268	659.6	65.55 to 1254	0.0283	*
7-8 min					
	Saline vs. MK-801	-797.5	-1353 to -242.1	0.0063	**
	MK-801 vs. LY379268	731.3	80.50 to 1382	0.0264	*
8-9 min					
	Saline vs. MK-801	-775.2	-1298 to -252.4	0.0048	**
	MK-801 vs. LY379268	803.5	165.7 to 1441	0.0131	*
	MK-801 vs. LY379268 and MK801	703.7	40.36 to 1367	0.0375	*
9-10 min					
	Saline vs. MK-801	-835.6	-1290 to -381.1	0.0011	**
	MK-801 vs. LY379268	846	208.5 to 1484	0.0096	**
	MK-801 vs. LY379268 and MK801	596.3	37.85 to 1155	0.0361	*
10-11 min					
	Saline vs. MK-801	-896	-1500 to -291.7	0.0066	**
	MK-801 vs. LY379268	847.6	119.4 to 1576	0.0214	*
11-12 min					
	Saline vs. MK-801	-1043	-1720 to -366.3	0.006	**
12-13 min					
	Saline vs. MK-801	-923.8	-1453 to -394.3	0.003	**
	MK-801 vs. LY379268	767.8	83.77 to 1452	0.0266	*
13-14 min					
	Saline vs. MK-801	-878.3	-1373 to -383.4	0.0018	**
	MK-801 vs. LY379268	709.5	5.980 to 1413	0.0479	*
	MK-801 vs. LY379268 and MK801	674.9	38.92 to 1311	0.0374	*
14-15 min					
	Saline vs. MK-801	-676	-1180 to -172.1	0.0098	**
	MK-801 vs. LY379268	669.1	4.991 to 1333	0.0481	*
	MK-801 vs. LY379268 and MK801	671.4	131.9 to 1211	0.0153	*
